# *Saccharomyces cerevisiae* oral immunization in mice using multi-antigen of the African swine fever virus elicits a robust immune response

**DOI:** 10.3389/fimmu.2024.1373656

**Published:** 2024-04-29

**Authors:** Shuo Gao, Wenfeng Zuo, Chao Kang, Zhong Zou, Kaiqi Zhang, Jun Qiu, Xiaomin Shang, Jingjing Li, Yuanfeng Zhang, Qi Zuo, Ya Zhao, Meilin Jin

**Affiliations:** ^1^ College of Veterinary Medicine, Huazhong Agricultural University, Wuhan, China; ^2^ State Key Laboratory of Agricultural Microbiology, Huazhong Agricultural University, Wuhan, China; ^3^ Research Institute of Wuhan Keqian Biology Co., Ltd, Wuhan, China; ^4^ College of Veterinary Medicine, Henan Agricultural University, Zhengzhou, China; ^5^ College of Animal Sciences, Yangtze University, Jingzhou, Hubei, China

**Keywords:** African swine fever virus, *Saccharomyces cerevisiae*, oral immunization, δ-integration, mucosal immune responses, cellular immune responses

## Abstract

African swine fever virus (ASFV) is one of the most complex viruses. ASFV is a serious threat to the global swine industry because no commercial vaccines against this virus are currently available except in Vietnam. Moreover, ASFV is highly stable in the environment and can survive in water, feed, and aerosols for a long time. ASFV is transmitted through the digestive and respiratory tract. Mucosal immunity is the first line of defense against ASFV. *Saccharomyces cerevisiae* (SC), which has been certified by the U.S. Food and Drug Administration and has a generally recognized as safe status in the food industry, was used for oral immunization in this study. ASFV antigens were effectively expressed in recombinant SC strains with high DNA copy numbers and stable growth though surface display technology and chromosome engineering (δ-integration). The recombinant SC strains containing eight ASFV antigens—KP177R, E183L, E199L, CP204L, E248R, EP402R, B602L, and B646L— induced strong humoral and mucosal immune responses in mice. There was no antigenic competition, and these antigens induced Th1 and Th2 cellular immune responses. Therefore, the oral immunization strategy using recombinant SC strains containing multiple ASFV antigens demonstrate potential for future testing in swine, including challenge studies to evaluate its efficacy as a vaccine against ASFV.

## 1 Introduction

African swine fever (ASF) is an acute, febrile, highly contagious infectious disease caused by the African swine fever virus (ASFV) and affects pigs and wild boars (1). ASFV has high morbidity and mortality, posing a significant threat to the global pig industry (2). ASFV, from the Asfarviridae family, is a large (250-260 nm in diameter) icosahedral virus with a multi-layer coating structure and double-stranded DNA (3). ASFV is the only known DNA virus transmitted by arthropods (4). This virus has various components, including a genome, nucleocapsid, inner capsid, and outer capsid. Further, the virus contains at least 160 open reading frames and more than 160 proteins (5). However, given the genomic complexity, unique biological characteristics, and our limited understanding of ASFV, the development of effective vaccines has faced significant obstacles (6, 7). The first reported case of ASF occurred in Kenya in 1921, and ASFV spread to Europe in 1960. ASFV was detected in the Russian Federation in 2007, China in 2018, and other Asian countries, including Vietnam, Mongolia, and Cambodia, in subsequent years (4, 8–10). Given the serious threat to the pig industry, food security, and economic trade, ASF has been listed as a notifiable animal disease by the World Organization for Animal Health, and China categorizes it as a category I animal disease (11, 12). Thus, developing safe and effective vaccines against ASFV is essential.

ASFV-inactivated vaccines generate high levels of antibodies but offer limited immune protection when prepared using traditional methods. Additionally, the use of adjuvants did not improve the efficiency of ASFV-inactivated vaccines (13). ASFV vaccines are typically produced from live attenuated viruses, protein subunits, or recombinant vectors (14, 15). Live attenuated ASFV vaccines require robust safety testing, before being considered safe for widespread use (16–19). Subunit vaccines have limited efficacy because of the lack of effective delivery systems and knowledge about protective antigens (20, 21). A previous study demonstrated that a mixture of eight viral antigens protected pigs against a lethal dose of ASFV (22). Moreover, combined immunization (intramuscular injection + nasal immunization) with five viral vector antigens protected pigs against virulent ASFV strains (11). While there are currently no commercial vaccines against ASFV available except in Vietnam (23), these successful cases demonstrate that cellular and humoral immune responses induced by recombinant vector-based ASFV antigen vaccines can effectively protect hosts. Additionally, mucosal immune defenses triggered by oral immunization can prevent virus invasion and secondary transmission.

As an expression host, the eukaryotic yeast *Saccharomyces cerevisiae* (SC) possesses post-translational modifications and can tolerate harsh environmental conditions (24). SC is easy to manipulate genetically, decreasing production costs. Additionally, SC has been approved for safety by the U.S. Food and Drug Administration (FDA) and holds a generally recognized as safe (GRAS) status in the food industry. The recombinant SC strains allows the production of highly glycosylated foreign proteins. This glycosylation process can lead to MHC-restricted antigen presentation in mammals, activating T lymphocytes (25). SC activates CD8+ cells via antigen cross-presentation, stimulating antigen-specific cell killing (26). Additionally, the cell wall of SC contains β-glucan and mannan, which are excellent adjuvants. Specifically, β-glucan targets immune cells through pathogen-associated molecular patterns, activating immune cells to regulate immune responses (27). Mannan promotes the maturation and differentiation of dendritic cells. Moreover, mannan binds to complement receptors and facilitates macrophage phagocytosis, enhancing the cytotoxicity of natural killer cells (28). SC interacts with the mannan receptor (CD206) on dendritic cells, enhancing the ability of these cells to recognize and present antigens (29). One of the primary considerations when expressing heterologous antigens in yeast is the selection of suitable vectors. Conventional episomal plasmids in SC can maintain 5 to 30 copy numbers, improving gene expression (30). However, these plasmids often lack stability and cannot be cultured for extended periods under non-selective conditions, hindering large-scale production (31). Moreover, the yeast genome contains 425 copies of the δ-sequence. Gene copy numbers can be increased using δ-sequences as target sites in the yeast genome for homologous recombination. Nevertheless, achieving high-copy integration and stable expression is challenging (32).

Dendritic cells (DCs) are crucial in pathogen recognition and activating adaptive immune responses due to their strong antigen-presenting ability (33). A targeting molecule called Dendritic cell targeting peptide (DCpep) enhances DCs’ efficiency in recognizing and taking up antigens, thus boosting the immune response (34). In gastrointestinal immunity, DCs located in the subepithelial dome (SED) region below the follicle-associated epithelium (FAE) play a significant role by directly capturing antigens from M cells (35–38). Immature DCs are highly efficient in antigen phagocytosis and mature into active DCs upon antigen uptake or specific stimuli. Recognition of pathogen-associated molecular patterns (PAMP) or damage-associated molecular patterns (DAMP) by pattern recognition receptors (PRRs) triggers DC activation and maturation (39). Upon reaching a lymph node, immature DCs mature further and can influence adaptive immunity by upregulating antigen presentation mechanisms, including MHC-II, costimulatory molecules, and pro-inflammatory cytokines (40, 41). These mature DCs then migrate to the T-cell area of lymphoid tissue to stimulate antigen-specific T cells (39).

We previously identified several protective ASFV antigens through literature search. Of these, eight antigens—KP177R, E183L, E199L, CP204L, E248R, EP402R, B602L, and B646L—were strongly implicated in the adsorption, internalization, endocytosis, and membrane fusion of ASFV (3, 42–45). However, most of these proteins possess transmembrane regions, nuclear localization signals, or endoplasmic reticulum localization signals, hindering expression on the surface of SC strains. To overcome this limitation, we performed biological analysis and immunological identification of the antigenic regions of these proteins. In this paper, we selected eight truncated antigenic regions. By δ-integration and high-throughput screening, we successfully obtained eight recombinant SC strains that efficiently expressed ASFV antigens on the cell surface. Furthermore, we conducted an evaluation of immunogenicity and antigenic competition in orally immunized mice with these recombinant SC strains, aiming to determine their potential for future challenge testing in swine.

## 2 Materials and methods

### 2.1 Ethical statement

The animal studies were approved by the Ministry of Agriculture and Rural Affairs of China and the Animal Care and Use Committee of Huazhong Agriculture University (HZAUSW-2022–0030). Every effort was made to minimize animal pain, suffering, and distress and to reduce the number of animals used.

### 2.2 Yeast strains, media, and culture conditions

The SC strain EBY100, commonly used as a yeast surface display system, was plated on yeast extract peptone dextrose (YPD) agar medium (20 g/L glucose, 20 g/L tryptone, and 10 g/L yeast cell extract [Sigma]). Plasmid vectors were transformed into host yeast cells using the lithium acetate method, as described previously (32). SC transformants were selected on synthetic minimal SD medium supplemented with auxotrophic requirements. Single transformants were plated on YPD medium and cultured in an incubator at 30°C for 48 h. Genomic DNA was analyzed by PCR using a yeast colony PCR kit (Weidi Bio, Shanghai, China) and a vector universal primer pair (F:5`-CCCAGGCTTTACACTTTATGCTT-3`, R: 5`-GCCAGCTTTTGTTCCCTTTAGTGAG-3`) to confirm correct recombination. PCR products were separated on a 1% (w/v) agarose/TAE gel. The expression of recombinant proteins was induced at 30°C for 48 h. After induction, all recombinant SC strains were preserved in 50% glycerol and stored at −80°C until use.

### 2.3 Construction of EBY100/pTy1E−ASFV antigens

The amino acid sequences of KP177R, E183L, E199L, CP204L, E248R, EP402R, B602L, and B646L were obtained based on the genome sequence of the ASFV HLJ strain (MK333180). The antigen structure, hydrophilicity, and epitopes were predicted and analyzed to select the optimal antigen region for gene synthesis. Codon optimization and ASFV gene synthesis were performed by GenScript Biotech Corporation. The synthesized genes were cloned into pUC57, and the sequence was confirmed by sequencing. The genes encoding KP177R, E183L, E199L, CP204L, E248R, EP402R, B602L, and B646L were linked in tandem and subcloned into the yeast expression vector pTy1E containing a V5 tag at the C-terminal using the NEBuilder HiFi DNA Assembly Master Mix (New England Biolabs, Inc.). The obtained plasmids—pTy1E-KP177R, pTy1E-E183L, pTy1E-E199L, pTy1E-CP204L, pTy1E-E248R, pTy1E-EP402R, pTy1E-B602L, and pTy1E-B646L—were linearized with *Eco*R1 (New England Biolabs, Inc.) to remove the antibiotic resistance gene. The linearized plasmids were used to transform the host strain EBY100.

### 2.4 Measurement of gene copy number by quantitative polymerase chain reaction

Total DNA was extracted from snap-frozen yeast cell samples using the VAMNE Magnetic Pathogen DNA Kit (Novizan, Nanjing, China). The copy number was determined by comparing the C_t_ values of target genes and internal reference genes, as described previously. A specific DNA fragment in the pTy1E plasmid was selected as the target gene, and ACT1 served as the internal reference. The qPCR standard curve was analyzed using the pTy1E plasmid and pMD18T plasmid (containing one copy of ACT1) as templates. The target gene was amplified using primer pair (F:5`-CCCAGGCTTTACACTTTATGCTT-3`, R: 5`-GCCAGCTTTTGTTCCCTTTAGTGAG-3`), while the internal reference gene was amplified using primer pair (F:5`-ATGTTTAGAGGTTGCTGCTTTGGTT-3`, R: 5`-TAGATGGGAAGACAGCACGAGGA-3`). qPCR analysis was conducted using the QuantStudio 6 system (Applied Biosystems, Waltham, MA, USA).

### 2.5 Analysis of genetic stability

Recombinant strains with different copy numbers were subcultured to ensure the growth stability and genetic stability of strains by δ-integration. For that purpose, strains were grown until OD_600_ reached 0.1, and 1% of the culture was transferred to 5 mL of YPD medium. Subsequently, the strains were subcultured 50 times. Strains from subcultures 1, 10, 20, 30, 40 and 50 were used as seeds for cultivation and analysis.

### 2.6 Measurement of ASFV antigen expression

Recombinant yeast strains expressing different antigens were used in the experiments. The pellet of approximately 10^7^ cells (OD_600 _= 1) was collected 48 h after induction. The pellet was washed thrice with 500 µL of PBS for subsequent analysis using Western blotting, indirect immunofluorescence, and flow cytometry.

For Western blotting, proteins were analyzed by SDS-PAGE and transferred to nitrocellulose membranes. The membranes were blocked with TBST containing 5% skim milk powder and 2% bovine serum albumin (BSA) for 2 h at room temperature. Then, the blots were incubated with anti-V5 monoclonal antibody (Biodragon, Beijing) overnight at 4°C, followed by incubation with horseradish peroxidase (HRP)-labeled goat anti-mouse IgG (Biodragon, Beijing) for 1 h at room temperature. Immunoreactive bands were visualized using an enhanced chemiluminescence system (Advansta, San Jose, CA, USA). For indirect immunofluorescence and flow cytometric analyses, SC cell pellet samples were blocked with 5% BSA at 30°C for 2 h and then incubated with anti-V5 monoclonal antibody at 30°C for 2 h, followed by incubation with DyLight 488-labeled goat anti-mouse IgG (Biodragon, Beijing) for 90 min at 30°C. Then, 5 µL of SC cells were used in immunofluorescence assays (Olympus, Tokyo, Japan), and 300 µL of yeast cells were analyzed by flow cytometry (Agilent, United States).

### 2.7 Mouse vaccination

Eight-week-old female BALB/c mice were randomly divided into 10 groups of five mice. One group was orally immunized with EBY100 (2.0 × 10^9^ CFU) and served as a control. Immunization was performed every 14 days, with a total of three immunizations. Mouse serum and fecal samples were collected 14, 28, and 42 days after the first immunization. On day 42, the mice were euthanized, and serum samples and spleen cells were collected for analysis by ELISA, flow cytometry, and ELISpot.

### 2.8 Quantification of ASFV-specific IgG and IgA titers by ELISA

Antigen-specific IgG and IgA antibodies in mouse serum and stool were quantified by ELISA. In brief, a 96-well plate was coated with 100 ng of KP177R, E183L, E199L, CP204L, E248R, EP402R, B602L, or B646L protein (Wuhan Keqian Biology Co., Ltd., Wuhan, China) overnight at 4°C. The plate was blocked with PBST and 5% skim milk for 2 h at room temperature. Serially diluted serum samples or fecal samples were added to each well, followed by incubation at 37°C for 2 h. HRP-conjugated goat anti-mouse IgG (Abcam, ab97023), IgG1 (Abcam, ab97240), IgG2a (Abcam, ab97245), or IgA (Abcam, ab97235) antibodies were added to the wells, followed by incubation at 37°C for 1 h. The assays were performed using 3,3′,5,5′-tetramethylbenzidine as the colorimetric substrate, and the optical density was measured at 450 nm. The cut-off value was determined by calculating the OD_450 _+ 3 SDs of serum samples from unvaccinated animals. The endpoint titer was calculated as the reciprocal of the highest serum dilution at which OD_450_ was equal to or greater than the cut-off value.

### 2.9 Analysis of cellular immune responses induced by recombinant SC strains-immunized mice using ELISpot and flow cytometry

ELISpot assays were conducted using freshly isolated mouse spleen lymphocytes to evaluate antigen-specific cellular immunity using IFNγ- or IL-4-secreting cells. Sterile 96-well microtiter plates were activated in RPMI-1640 and precoated with mouse IFN-γ (MabTech, 3321-4AST-2) or IL-4 (MabTech, 3311-4APW-2). Spleen lymphocytes were isolated using mouse lymphocyte separation medium (Dakewei, Beijing, China, 7211011), and 5.0 × 10^5^ cells were transferred to each well. Cells were stimulated with 10 μg/ml of KP177R, E183L, E199L, CP204L, E248R, EP402R, B602L, or B646L for 30 h. The plates were incubated with streptavidin-alkaline phosphatase conjugate (1:1000) diluted in PBS containing 0.5% fetal bovine serum for 1 h, and the reaction was developed with nitroblue tetrazolium bromochloroindolyl phosphate toluidine salt. Spots were counted using an automated ELISpot plate reader (IRIS, Mabtech, Sweden) and Apex software (Mabtech) with default settings. Spleen lymphocytes (5.0 × 10^5^) were collected from each group and transferred to test tubes. The cells were incubated with fluorescein isothiocyanate-conjugated hamster anti-mouse CD3e (Cat. #553061, BD Biosciences, San Jose, CA, USA), phycoerythrin-conjugated rat anti-mouse CD8a (Cat. #553032, BD Biosciences), and allophycocyanin-conjugated rat anti-mouse CD4 (Cat. #553051, BD Biosciences). Cells were analyzed by flow cytometry (Agilent, United States), and data were analyzed using FlowJo software version 10.6.1 (BD Biosciences).

### 2.10 Statistical analysis

Data are presented as means ± standard deviations (SDs). All statistical analyses were performed using GraphPad Prism version 9.4.1.681 (La Jolla, CA, USA). Antibody titers and IFN-γ and IL-4 levels were determined by two-way analysis of variance followed by the least significant difference or Tukey’s test. Survival was calculated using the Kaplan-Meier method. Data are shown as the mean and standard deviation (mean ± SD). *p < 0.05; **p < 0.01; ***p < 0.001.

## 3 Results

### 3.1 Recombinant SC strains were constructed by δ-integration to achieve a high copy number of ASFV antigens

**Figure 1:**
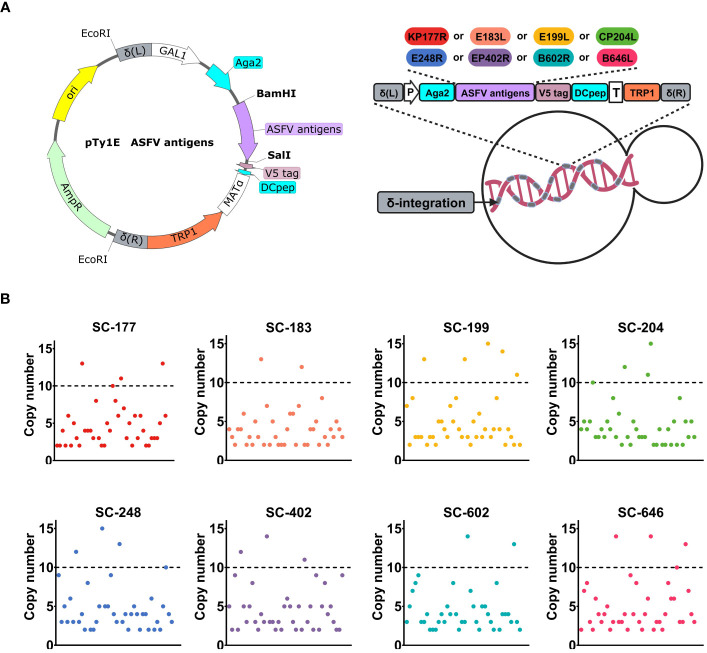
Recombinant Saccharomyces cerevisiae strains were constructed by δ-integration to achieve a high copy number of African swine fever virus (ASFV) antigens. **(A)** Schematic diagram of the construction of an ASFV antigen-based recombinant SC strains. The open-reading frame included Aga2, one ASFV antigen (KP177R, E183L, E199L, CP204L, E248R, EP402R, B602L, or B646L), V5 tag, and Dcpep. P is the GAL1 promoter, and T is the MATα terminator. The TRP1 auxotrophic screening marker with truncated promoter is indicated in orange. δ-integration sites are indicated in gray. **(B)** High-throughput screening of recombinant strains with high copy numbers. The copy number of each recombinant strain expressing one antigen (SC-177, SC-183, SC-199, SC-204, SC-248, SC-402, SC-602, or SC-646) is shown in different colors.

To develop a safe and effective oral immunization strategy, the SC strain EBY100, with GRAS status in the food industry, was utilized as the starting strain. The structure, hydrophilicity, and epitopes of KP177R, E183L, E199L, CP204L, E248R, EP402R, B602L, and B646L were predicted and analyzed to obtain the truncated sequences of antigen genes. Then, these antigen sequences were codon optimized for expression in SC. A recombinant strain that contained ASFV antigens on its surface and could be utilized in large-scale industrial production was obtained using the procedure outlined in **Figure 1A** and described in the Methods section.

We designed eight integration cassettes that contained the gene of interest (ASFV antigen gene V5-DCpep) along with genes encoding auxotrophic screening markers (TRP1 with truncated promoter). These integration cassettes were flanked by the left and right homology arms of the δ sequence. This strategy capitalizes on the presence of multiple copies of the δ sequence and improves recombination in SC under auxotrophic stress. These integration cassettes were inserted into the chromosomes of eight EBY100 strains: SC-177, SC-183, SC-199, SC-204, SC-248, SC-402, SC-602, and SC-646. Forty transformants were analyzed by qPCR to identify and select recombinant strains with high copy number of surface-expressed ASFV antigens. The tryptophan auxotrophy necessary for transformation using these expression cassettes was achieved by deleting the first 62 bases of the TRP1 promoter. Each recombinant strain yielded two to five transformants with a copy number of 10 or more (**Figure 1B** and **Supplementary Figure 1A**). The transformants that passed the screening tests for growth potential, genetic stability, and stable antigen expression were selected for further experiments.

### 3.2 The antigen mixture was efficiently and stably expressed on the surface of the EBY100 strain

**Figure 2:**
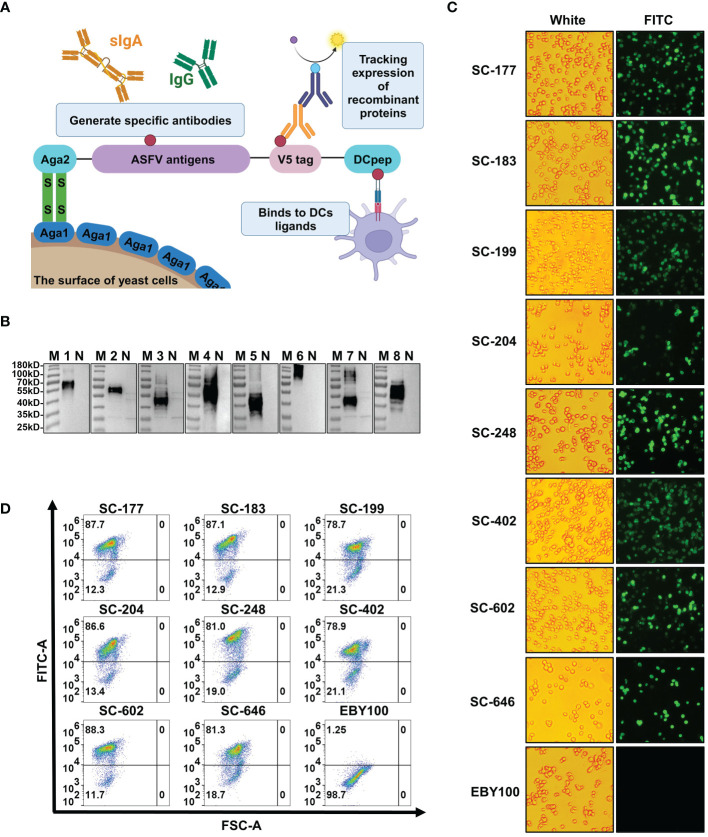
African swine fever virus (ASFV) antigens are efficiently and stably expressed on the surface of the EBY100 *Saccharomyces cerevisiae* (SC) strain. **(A)** Schematic diagram of recombinant yeast strains expressing ASFV antigens. **(B)** Western blotting analysis of the expression of Aga2-ASFV antigens in each recombinant SC strain. M is molecular weight markers. N, 1, 2, 3, 4, 5, 6, 7, and 8 represent SC strains EBY100, SC-177, SC-183, SC-199, SC-204, SC-248, SC-402, SC-602, and SC-646. **(C)** Indirect immunofluorescence of recombinant strains. Cells were seeded on 96-well cell culture plates, and the presence of ASFV antigens was analyzed by inverted fluorescence microscopy. **(D)** The level of antigen expression in each strains was determined by flow cytometry.

A thorough screening was performed to identify strains with high DNA copy number, high growth potential, and genetic stability (**Supplementary Figures 1B, C**). We compared the growth curve (**Supplementary Figure 1B**) of each strain with that of the EBY100 strain and performed PCR detection (**Supplementary Figure 1C**) of the target gene of the recombinant yeast for 50 consecutive generations. Eight recombinant strains—SC-177, SC-183, SC-199, SC-204, SC-248, SC-402, SC-602, and SC-646—were selected based on these criteria. Each strain was engineered to express one of eight ASFV antigen—KP177R, E183L, E199L, CP204L, E248R, EP402R, B602L, and B646L—on the cell surface. The a-lectin protein subunits aga1 and aga2 are present on the yeast cell wall surface. When the aga2-fusion protein is induced and expressed, it forms a disulfide bond with aga1 on the cell wall surface upon secretion (**Figure 2A**) (46). The V5 tag on the target gene’s C-terminus can be detected by western blotting, indirect immunofluorescence, and flow cytometry. The V5 tag is followed by the 12-amino acid dendritic cell-targeting peptide DCpep (FYPSYHSTPQRP), which plays a significant role in mucosal immunity (47, 48). Western blots revealed that Aga2-fusion proteins were absent from all negative controls but were present in all recombinant strains (**Figure 2B**). Based on glycosylation prediction, the molecular weight of the target band was larger than the theoretical molecular weight because of N-glycosylation modification, consistent with the surface expression of target proteins in recombinant strains. Furthermore, indirect immunofluorescence microscopy revealed the presence of green fluorescent protein in the experimental groups but not in the EBY100 negative control (**Figure 2C**). Aga2-ASFV antigens had increased fluorescence, indicating high antigen expression on the surface of recombinant strains. These results are consistent with flow cytometry data (**Figure 2D**). Compared with the control strain, the percentage of expression of ASFV surface antigens in the recombinant strains was 78.7-88.3%.

### 3.3 The ASFV antigen-based oral immunization strategy induced high-level and balanced antigen-specific serum IgG and mucosal IgA immune responses

**Figure 3:**
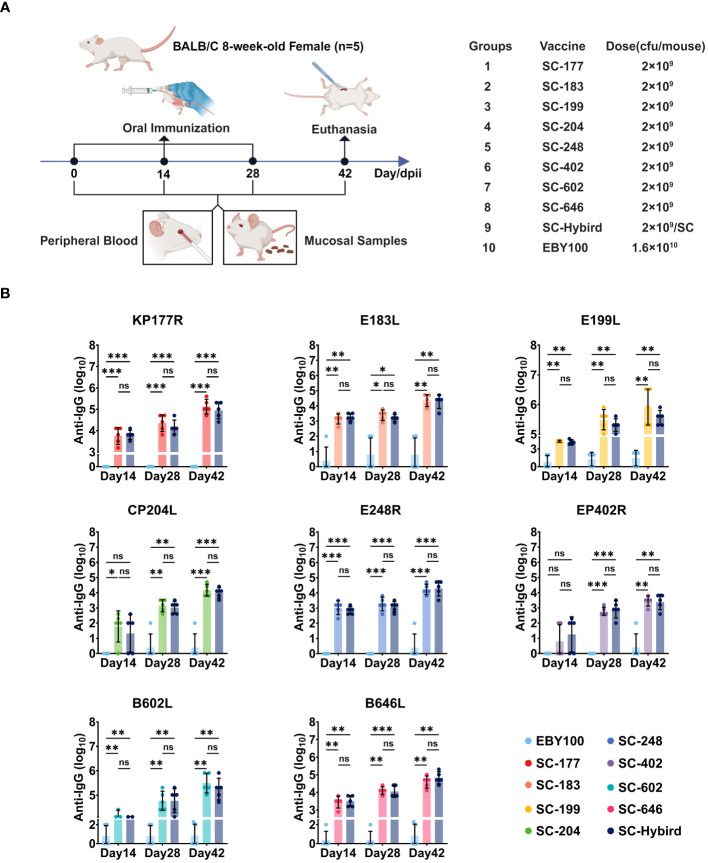
The recombinant *Saccharomyces cerevisiae* (SC) strains containing African swine fever virus (ASFV) antigens induces high-level antigen-specific serum IgG immune responses. **(A)** Experimental design. Eight-week-old BALB/c mice were orally inoculated with recombinant SC strains or EBY100 strain. Peripheral blood and feces were collected at different time points. The mice were sacrificed 42 days post-primary immunization (dppi). Spleens were harvested, and lymphocytes were collected. **(B)** Reciprocal of recombinant SC strains-induced serum IgG titers. Humoral immune responses to antigens KP177R, E183L, E199L, CP204L, E248R, EP402R, B602L, or B646L in the groups immunized with a single antigen or an antigen mixture compared with the EBY100 group at 14, 28, and 42 dppi. * p < 0.05; ** p < 0.01; *** p < 0.001; not significant (ns) p≥0.05.

Vaccines containing one or two ASFV antigens do not offer substantial protection to recipients. Conversely, multi-antigen vaccines can elicit strong immune responses, providing high protection (22). However, antigen cocktails may result in antigenic competition (11). To evaluate the effects of recombinant SC strains on the induction of humoral, mucosal, and cellular immunity, the strains were administered orally to 8-week-old female BALB/c mice on 0, 14, and 28 days post-primary immunization (dppi). The mice were randomly divided into 10 groups of five animals (**Figure 3A**). Groups 1 to 8 (designated single strain [SS]) were orally immunized with SC-177, SC-183, SC-199, SC-204, SC-248, SC-402, SC-602, or SC-646, respectively, with 2.0 × 10^9^ colony-forming units (CFU) of each strain per mouse. Group 9 (designated multiple strains [MS]) was orally immunized with eight strains (2.0 × 10^9^ CFU of each strain per animal). Group 10 (control) was orally immunized with 1.6 × 10^10^ CFU of the EBY100 strain. To quantify the levels of antibodies generated by humoral and mucosal immune responses induced by the recombinant SC strains, ELISA was performed using recombinant proteins as coating antigens. Serum and fecal samples were collected from mice vaccinated at 0, 14, 28, and 42 dppi. The levels of serum IgG titers and fecal IgA titers against specific antigens were determined by ELISA (**Figure 3B**). The antigen-specific serum IgG antibody titers were significantly higher (P**<**0.05) in the SS groups than in the EBY100 group at 14 dppi, except for SC-402 (P≥0.05). A possible explanation for this result is that some mice did not develop a strong immune response against EP402R. The antigen-specific IgG titers elicited by the recombinant SC strains increased as the number of immunizations increased. At 28 and 42 dppi, mice in all SS groups had acquired high levels of ASFV-specific antibody titers (P**<**0.05). In addition, serum IgG antibody levels were similar between the SS and MS groups (P≥0.05), suggesting that the AM can induce a balanced immune response without antigenic competition.

**Figure 4:**
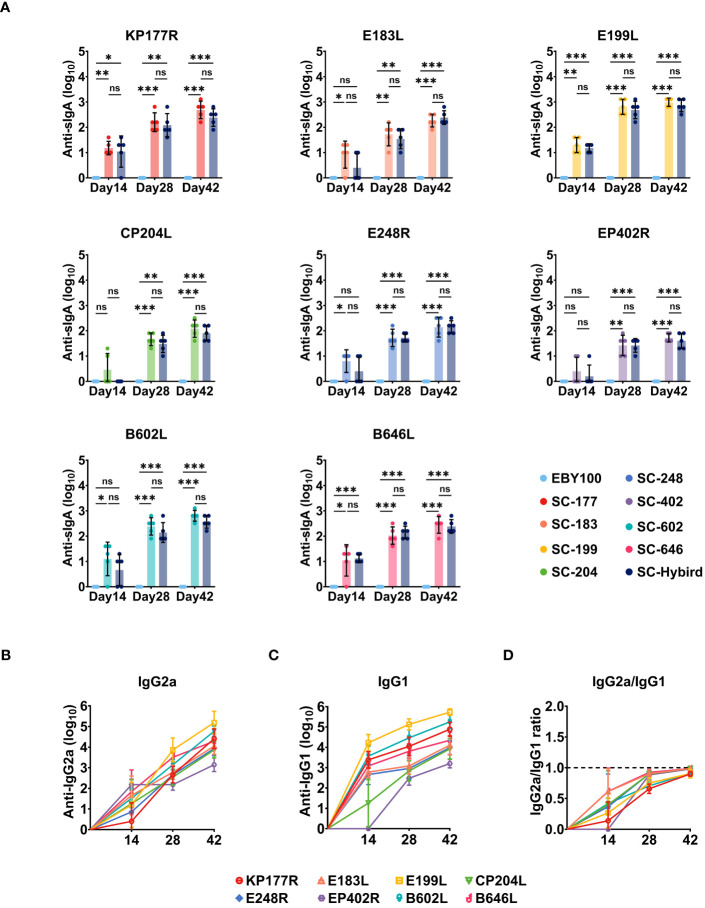
The recombinant *Saccharomyces cerevisiae* (SC) strains containing African swine fever virus (ASFV) antigens induces high-level and balanced antigen-specific serum IgG and mucosal IgA immune responses in mice. **(A)** Reciprocal of ASFV antigen-specific fecal IgA titers induced by the recombinant SC strains. Mucosal immune responses to antigens KP177R, E183L, E199L, CP204L, E248R, EP402R, B602L, or B646L in the groups immunized with a single antigen or an antigen mixture compared with the EBY100 group at 14, 28, and 42 days post-primary immunization. **(B–D)** Reciprocal of recombinant SC strains-induced serum IgG2a, IgG1, and IgG2a/IgG1 titers. * p < 0.05; ** p < 0.01; *** p < 0.001; not significant (ns) p≥0.05.

Mucosal immunity protects the body from foreign pathogens, serving as the primary barrier against invasion (49). This defense mechanism is particularly effective in preventing disease emergence and spread. For oral immunization strategy, the immune response occurs in the intestinal mucosa. Intestinal antigens induce the activation and proliferation of lymphocytes, which differentiate and multiply in local lymph nodes. Then, lymphocytes migrate to the bloodstream during homing and eventually return to the intestinal mucosa (49). To evaluate the efficacy of mucosal immune defenses, we measured ASFV-specific IgA titers in the feces (**Figure 4A**). At 14 dppi, the IgA antibody titers against KP177R, E183L, E199L, E248R, B602L, and B646L were significantly higher in the SS group than in the EBY100 group (P**<**0.05). Consistent with serum IgG responses, ASFV-specific IgA antibody titers increased with the number of immunizations in all SS groups. Moreover, fecal IgA responses were similar between the SS and MS groups (P≥0.05). The IgG2a/IgG1 ratio is an indicator of Th2/Th1-mediated responses. We measured ASFV-specific IgG2a and IgG1 titers at 0, 14, 28, and 42 dppi in the MS group and calculated IgG2a to IgG1 ratios (**Figures 4B–D**). At 14 dppi, ASFV-specific IgG1 titers were higher than ASFV-specific IgG2a titers (**Figures 4B, C**), suggesting that Th2 responses predominated in the early stage of immunity. However, the IgG2a to IgG1 ratio approached 1 as the number of immunizations increased (**Figure 4D**), indicating that the recombinant SC strains induced Th1 and Th2 responses. The oral immunization strategy effectively stimulated humoral and systemic mucosal immunity against KP177R, E183L, E199L, CP204L, E248R, EP402R, B602L, and B646L. Additionally, immunization with the AM triggered Th1 and Th2 immune responses without causing antigenic competition.

### 3.4 The ASFV multiple-antigen recombinant SC strains elicited robust antigen-specific cellular immune responses

**Figure 5:**
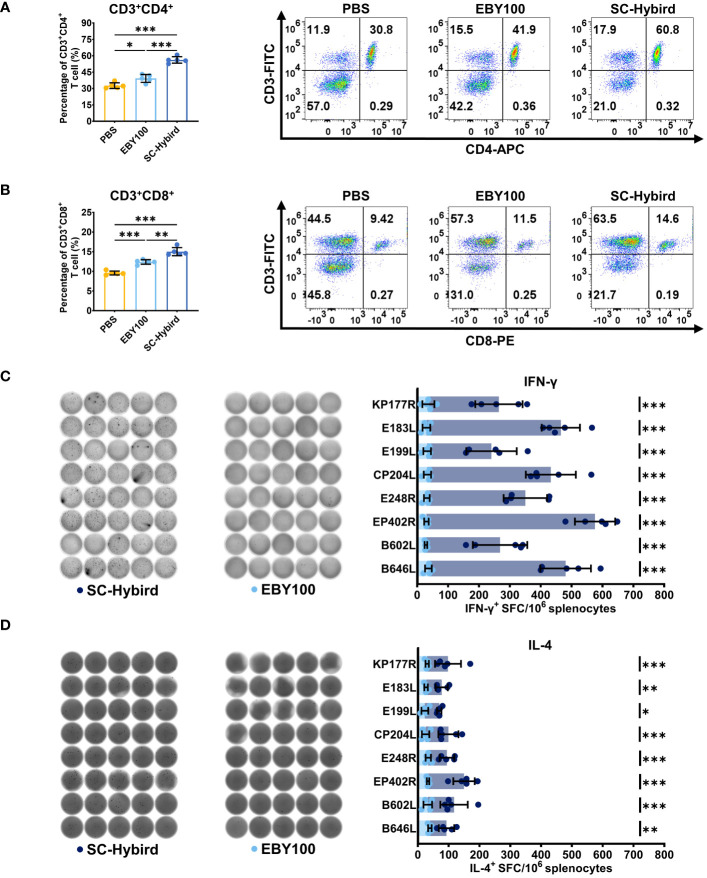
A recombinant *Saccharomyces cerevisiae* strains containing African swine fever virus (ASFV) antigens elicits robust antigen-specific cellular immune responses in mice. **(A, B)** Flow cytometry analysis of the percentage of splenic CD4+ or CD8+ T cells. **(C, D)** The response of different groups after *in vitro* stimulation, IFN-γ and IL-4-producing T cells and statistical analysis. Images were acquired and quantified using a microplate reader. * p < 0.05; ** p < 0.01; *** p < 0.001.

The dietary supplementation of SC enhances the immune response of animals by promoting lymphocyte proliferation and differentiation (25, 31). Spleen lymphocytes from mice in the MS, EBY100, or PBS groups were isolated at 42 dppi. The proliferation of CD3+, CD4+ and CD8+ T cells in the spleen was analyzed by flow cytometry (**Figures 5A, B**). Compared with the PBS group, the number of activated CD3+CD4+ T cells and CD3+CD8+ T cells was significantly higher (P**<**0.05) in the EBY100 group, which did not carry foreign antigens. This finding supports the effectiveness of SC as a feed additive. The number of CD3+CD4+ T cells and CD8+ T cells was higher in the MS group than in the EBY100 and PBS groups (P**<**0.05). Furthermore, the percentage increase of CD4+ T cells was higher than the percentage increase of CD8+ T cells, the cellular immunity induced by the AM is more inclined toward helper T cell immunity responses. In conclusion, immunization with the AM resulted in the percentage increase of CD4+ and CD8+ T cell. Previous studies have demonstrated that ASFV replicates in monocytes and macrophages, leading to high viral loads in multiple tissues and organs via the bloodstream (50). The activation of virus-specific cellular immune responses helps control ASFV infections (51). This study used enzyme-linked immunospot assay (ELISpot) to investigate the impact of the AM on memory T cells (**Figures 5C, D**). First, we stimulated spleen lymphocytes from mice in the MS and EBY100 groups with KP177R, E183L, E199L, CP204L, E248R, EP402R, B602L, or B646L. Then, we measured the levels of IFN-γ and IL-4 cytokines in lymphocyte supernatants. The levels of IFN-γ (**Figure 5C**) and IL-4 (**Figure 5D**) were significantly higher (P**<**0.05) in lymphocytes from the MS group upon stimulation with different antigens than the control antigen. IFN-γ levels were higher than IL-4 levels, suggesting that the AM induces Th1 and Th2 immune responses, with a preference for the former. Moreover, flow cytometry and ELISpot results indicated that the AM effectively induced a strong ASFV-specific cellular immune response.

## 4 Discussion

ASFV, one of the most complex viruses known to date, poses a serious threat to the global swine industry. No commercial vaccine against ASFV is currently available, except in Vietnam (23). Thus, ASF outbreaks are mitigated by limiting pig movement and mass-culling infected herds (5–7, 52). Disease-free countries prioritize the prevention of ASFV infections (53, 54). ASFV is highly stable in the environment and can survive in meat and blood for several months at room temperature. Moreover, this virus withstands extreme temperatures and pH (52). ASFV is transmitted through the oral ingestion of contaminated water, feed, or aerosols. The two main routes of infection are the digestive and respiratory tract (11). Mucosal immunity is the first line of defense against ASFV, providing a robust defense against infection and transmission. Therefore, developing an oral immunization strategy for ASFV is reasonable and feasible.

The mucosal immune system comprises gut-associated and nasal-associated lymphoid tissues, which provide strong protection against microbial infections (55). While most pathogens enter the body through mucosal surfaces, injection route primarily target the peripheral immune system (56). The peripheral immune system—bone marrow, spleen, and lymph nodes—combats invading pathogens and prevents further invasion. In contrast, the mucosal immune system acts as a barrier, preventing pathogen entry. Injectable route effectively stimulate the peripheral immune system; nonetheless, their ability to induce strong mucosal immune responses is unknown. Some oral vaccines, particularly those administered via the digestive or respiratory tract, are more effective than injectable vaccines. For instance, the injectable *Vibrio cholerae* vaccine triggers a weak immune response (49). In turn, the oral vaccine for cholera generates adequate group protection (57). Various oral vaccines have been successfully used to prevent human and animal diseases (48, 56, 58–61).

SC has a higher ability to express viral antigen proteins than prokaryotes (62). Moreover, SC has been certified as safe by the U.S. FDA and holds a GRAS status in the food industry, making it an ideal platform for the research and development of oral immunization strategy. The large-scale production of low-cost recombinant SC strains is crucial for preventing and controlling ASFV infections. The chromosomal engineering method based on δ-integration is widely used in yeast research. However, combining high-copy integration and stable expression is challenging (31, 32). This study successfully obtained ASFV multi-antigen recombinant SC strains by δ-integration and high-throughput screening. The recombinant SC strains exhibited excellent growth, genetic stability, and expression stability. Given the numerous potential protective antigens of ASFV, it is generally believed that immunization with multiple antigens can effectively prevent and control ASFV infections. However, antigen cocktails may cause antigenic competition (11, 22). This study demonstrated that the AM-based recombinant SC strains expressing antigens KP177R, E183L, E199L, CP204L, E248R, EP402R, B602L, and B646L on the surface of SC cells did not induce antigenic competition and stimulated strong immune responses.

The surface of lymphocytes in gut-associated and nasal-associated lymphoid tissues possess unique adhesion molecules, enabling these cells to re-enter mucosal sites. Upon activation by mucosal antigens, lymphocytes undergo differentiation and proliferation in local lymph nodes by homing. Then, lymphocytes enter the bloodstream through the thoracic duct and return to the mucosa. Mucosal tissues exhibit immunological interactions and are interconnected through a common membrane immune mechanism. Nasal and oral immunizations protect the reproductive mucosa against herpes and papillomavirus infections (63, 64). In line with these findings, our results showed that the oral administration of the multi-antigen recombinant SC strains induced systemic immune responses, including the generation of high levels of antigen-specific IgG titers in peripheral blood and secretory IgA titers in feces. Additionally, the recombinant SC strains promoted the development of a substantial and balanced population of B cells producing specific IgG1 and IgG2a antibodies. The coordinated response between the peripheral and mucosal immune systems is essential for combating ASFV infections.

The mucosal surface area is extensive and is the primary entry point for most pathogens. To prevent the invasion of pathogenic bacteria, approximately 80% of the immune cells in the immune system are concentrated in the mucosa (56), highlighting the crucial role of the mucosa in overall immunity. The most abundant antibody in mucosal secretions is secretory IgA. The daily production and secretion of IgA surpasses the combined levels of IgM and IgG. Notwithstanding, the primary effector cells on the mucosal surface are CD4+ and CD8+ T lymphocytes, not IgA + B cells (60). This fact suggests that mucosal immunity relies on T cell-mediated mechanisms and is not solely dependent on secreted antibodies.

Cellular immunity is crucial for generating memory and effector T cells through vaccination, resulting in long-lasting immune protection (65). The host responses to viral infections involve activating innate and adaptive immune systems (66). T cells strongly protect against viral infections. CD4+ T cells stimulate the production of antibodies by B cells and coordinate the responses of other immune cell types (67). CD4+ T cells initiate immune responses against infectious agents. Moreover, these cells differentiate into Th2 and Th1 cells that drive adaptive immune responses, while CD8+ T cells have cytotoxic activity. Thus, T lymphocytes can improve their cytotoxic activity by increasing the expression of CD8 proteins (68). CD8+ T cells primarily target infected cells and clear infections through pathways involving perforins, granzyme, and FasL (69). Compared with the PBS group, we found that the oral administration of the EBY100 strain improved cellular immune responses, evidenced by the increased proliferation of CD4+ and CD8+ T cells. Cellular immune responses may be nonspecific but enhance host resistance, confirming the excellent applicability of SC as a probiotic. The invasion and proliferation of pathogenic bacteria contribute to host death after ASFV infection (70, 71). Therefore, enhancing host immune resistance can reduce ASFV invasion. Additionally, CD4+ and CD8+ T cell proliferation was significantly higher in mice treated with the AM than in those treated with PBS or EBY100. This finding suggests that the T cell immunity triggered by the multi-antigen recombinant SC strains is based primarily on helper T cells, as evidenced by the higher proliferation of CD4+ T cells than CD8+ T cells.

Quantifying the expression of IFN-γ and IL-4 in spleen lymphocytes stimulated by the eight ASFV antigens allowed determining the type of immune response induced by the multi-antigen recombinant SC strains. Th1 cells secrete IL-2, IFN-γ, TNF-α, and other cytokines, mediating cellular immune responses (72). Th2 cells secrete IL-4, IL-5, IL-6, IL-10, and other cytokines, mediating humoral immune responses (73, 74). IFN-γ induced by Th1-type responses provide immune protection against ASFV (74–77). We observed that splenic lymphocytes stimulated by ASFV antigens increased IFN-γ concentrations, and IFN-γ levels were higher than IL-4 levels. Moreover, the IgG2a to IgG1 ratio supports the conclusion that immune responses caused by the multi-antigen recombinant SC strains are predominantly of the Th1 type.

Currently, there is no mouse vaccination model available for African Swine Fever Virus (ASFV). ASFV isolation, culture, and challenge studies require biosafety level 3 (BSL-3) facilities, which poses limitations on vaccine and drug development. ASFV is a highly complex virus that does not guarantee protection even with T cell and humoral responses post-vaccination (78, 79). Nevertheless, high antibody titers and cellular immune responses against multiple antigens may indicate the host’s ability to defend against viral infections. While mice are not the natural host for ASFV, they serve as a viable model for assessing antigen competition and immunogenicity. Our future research plans involve testing these recombinant SC strains in swine, the natural host of ASFV, and conducting challenge studies to assess their potential as ASFV vaccines.

In conclusion, the oral administration of the multi-antigen recombinant SC strains effectively induces ASFV-specific serum IgG and mucosal secretory IgA immune responses, activates CD8+ T cells, and stimulates a balanced immune response dominated by Th1 cells. Therefore, this recombinant SC strains, which is both safe and affordable, should be further evaluated in clinical trials.

## Data availability statement

Publicly available datasets were analyzed in this study. This data can be found here: NCBI Nucleotide repository, accession number MK333180.

## Ethics statement

The animal study was approved by The Animal Care and Use Committee of Huazhong Agriculture University. The study was conducted in accordance with the local legislation and institutional requirements.

## Author contributions

SG: Conceptualization, Data curation, Formal analysis, Investigation, Methodology, Software, Visualization, Writing – original draft, Writing – review & editing. WZ: Conceptualization, Formal analysis, Validation, Writing – review & editing. CK: Project administration, Supervision, Validation, Writing – review & editing. ZZ: Project administration, Supervision, Validation, Writing – review & editing. KZ: Data curation, Formal analysis, Investigation, Writing – review & editing. JQ: Data curation, Formal analysis, Investigation, Writing – review & editing. XS: Data curation, Formal analysis, Investigation, Writing – review & editing. JL: Data curation, Formal analysis, Investigation, Writing – review & editing. YFZ: Investigation, Writing – review & editing. QZ: Investigation, Writing – review & editing. YZ: Investigation, Writing – review & editing. MJ: Conceptualization, Funding acquisition, Methodology, Project administration, Resources, Writing – review & editing.

## References

[1] Karger A Perez-Nunez D Urquiza J Hinojar P Alonso C Freitas FB. An update on African swine fever virology. Viruses. (2019) 11:9–864. doi: 10.3390/v11090864 PMC6784044 31533244

[2] Sanchez EG Perez-Nunez D Revilla Y. Development of vaccines against african swine fever virus. Virus Res. (2019) 265:150–5. doi: 10.1016/j.virusres.2019.03.022 30922809

[3] Wang N Zhao D Wang J Zhang Y Wang M Gao Y. Architecture of african swine fever virus and implications for viral assembly. Science. (2019) 366:640–4. doi: 10.1126/science.aaz1439 31624094

[4] Gaudreault NN Madden DW Wilson WC Trujillo JD Richt JA. African swine fever virus: an emerging DNA arbovirus. Front Vet Sci. (2020) 7:215. doi: 10.3389/fvets.2020.00215 32478103 PMC7237725

[5] Forth JH Calvelage S Fischer M Hellert J Sehl-Ewert J Roszyk H. African swine fever virus - variants on the rise. Emerg Microbes Infect. (2023) 12:2146537. doi: 10.1080/22221751.2022.2146537 36356059 PMC9793911

[6] Dixon LK Sun H Roberts H. African swine fever. Antiviral Res. (2019) 165:34–41. doi: 10.1016/j.antiviral.2019.02.018 30836106

[7] Dixon LK Abrams CC Chapman DD Goatley LC Netherton CL Taylor G. Prospects for development of african swine fever virus vaccines. Dev Biol (Basel). (2013) 135:147–57. doi: 10.1159/000170936 23689892

[8] Zhao D Liu R Zhang X Li F Wang J Zhang J. Replication and virulence in pigs of the first african swine fever virus isolated in China. Emerg Microbes Infect. (2019) 8:438–47. doi: 10.1080/22221751.2019.1590128 PMC6455124 30898043

[9] Zakaryan H Revilla Y. African swine fever virus: current state and future perspectives in vaccine and antiviral research. Vet Microbiol. (2016) 185:15–9. doi: 10.1016/j.vetmic.2016.01.016 26931386

[10] Revilla Y Perez-Nunez D Richt JA. African swine fever virus biology and vaccine approaches. Adv Virus Res. (2018) 100:41–74. doi: 10.1016/bs.aivir.2017.10.002 29551143

[11] Liu W Li H Liu B Lv T Yang C Chen S. A new vaccination regimen using adenovirus-vectored vaccine confers effective protection against african swine fever virus in swine. Emerg Microbes Infect. (2023) 12:2233643. doi: 10.1080/22221751.2023.2233643 37401832 PMC10348041

[12] Shao Z Su S Yang J Zhang W Gao Y Zhao X. Structures and implications of the C962r protein of african swine fever virus. Nucleic Acids Res. (2023) 51:9475–90. doi: 10.1093/nar/gkad677 PMC10516667 37587714

[13] Blome S Gabriel C Beer M. Modern adjuvants do not enhance the efficacy of an inactivated african swine fever virus vaccine preparation. Vaccine. (2014) 32:3879–82. doi: 10.1016/j.vaccine.2014.05.051 24877766

[14] Liu Y Xie Z Li Y Song Y Di D Liu J. Evaluation of an I177l gene-based five-gene-deleted african swine fever virus as a live attenuated vaccine in pigs. Emerg Microbes Infect. (2023) 12:2148560. doi: 10.1080/22221751.2022.2148560 36378022 PMC9769145

[15] Yang S Miao C Liu W Zhang G Shao J Chang H. Structure and function of african swine fever virus proteins: current understanding. Front Microbiol. (2023) 14:1043129. doi: 10.3389/fmicb.2023.1043129 36846791 PMC9950752

[16] Liu L Wang X Mao R Zhou Y Yin J Sun Y. Research progress on live attenuated vaccine against african swine fever virus. Microb Pathog. (2021) 158:105024. doi: 10.1016/j.micpath.2021.105024 34089790

[17] Wang T Luo R Sun Y Qiu HJ. Current efforts towards safe and effective live attenuated vaccines against african swine fever: challenges and prospects. Infect Dis Poverty. (2021) 10:137. doi: 10.1186/s40249-021-00920-6 34949228 PMC8702042

[18] Barasona JA Cadenas-Fernandez E Kosowska A Barroso-Arevalo S Rivera B Sanchez R. Safety of african swine fever vaccine candidate lv17/wb/rie1 in wild boar: overdose and repeated doses. Front Immunol. (2021) 12:761753. doi: 10.3389/fimmu.2021.761753 34917082 PMC8669561

[19] Deutschmann P Forth JH Sehl-Ewert J Carrau T Viaplana E Mancera JC. Assessment of african swine fever vaccine candidate asfv-G-ΔMgf in a reversion to virulence study. NPJ Vaccines. (2023) 8:78. doi: 10.1038/s41541-023-00669-z 37248243 PMC10227017

[20] Cadenas-Fernandez E Sanchez-Vizcaino JM Kosowska A Rivera B Mayoral-Alegre F Rodriguez-Bertos A. Adenovirus-vectored african swine fever virus antigens cocktail is not protective against virulent arm07 isolate in eurasian wild boar. Pathogens. (2020) 9:3. doi: 10.3390/pathogens9030171 PMC7157622 32121082

[21] Zajac MD Trujillo JD Yao J Kumar R Sangewar N Lokhandwala S. Immunization of pigs with replication-incompetent adenovirus-vectored african swine fever virus multi-antigens induced humoral immune responses but no protection following contact challenge. Front Vet Sci. (2023) 10:1208275. doi: 10.3389/fvets.2023.1208275 37404778 PMC10316028

[22] Goatley LC Reis AL Portugal R Goldswain H Shimmon GL Hargreaves Z. A pool of eight virally vectored african swine fever antigens protect pigs against fatal disease. Vaccines (Basel). (2020) 8:2–234. doi: 10.3390/vaccines8020234 PMC7349991 32443536

[23] Borca MV Ramirez-Medina E Silva E Rai A Espinoza N Velazquez-Salinas L. Asf vaccine candidate asfv-G-ΔI177l does not exhibit residual virulence in long-term clinical studies. Pathogens. (2023) 12:6–805. doi: 10.3390/pathogens12060805 PMC10301618 37375495

[24] Bae JH Sung BH Kim HJ Park SH Lim KM Kim MJ. An efficient genome-wide fusion partner screening system for secretion of recombinant proteins in yeast. Sci Rep. (2015) 5:12229. doi: 10.1038/srep12229 26195161 PMC4508530

[25] Liu Z Zhou G Ren C Xu K Yan Q Li X. Oral administration of myostatin-specific recombinant saccharomyces cerevisiae vaccine in rabbit. Vaccine. (2016) 34:2378–82. doi: 10.1016/j.vaccine.2016.03.036 27005809

[26] Bernstein MB Chakraborty M Wansley EK Guo Z Franzusoff A Mostbock S. Recombinant saccharomyces cerevisiae (Yeast-cea) as a potent activator of murine dendritic cells. Vaccine. (2008) 26:509–21. doi: 10.1016/j.vaccine.2007.11.033 18155327

[27] Brown GD Taylor PR Reid DM Willment JA Williams DL Martinez-Pomares L. Dectin-1 is a major beta-glucan receptor on macrophages. J Exp Med. (2002) 196:407–12. doi: 10.1084/jem.20020470 PMC2193936 12163569

[28] Underhill DM Ozinsky A. Phagocytosis of microbes: complexity in action. Annu Rev Immunol. (2002) 20:825–52. doi: 10.1146/annurev.immunol.20.103001.114744 11861619

[29] Newman SL Holly A. Candida albicans is phagocytosed, killed, and processed for antigen presentation by human dendritic cells. Infect Immun. (2001) 69:6813–22. doi: 10.1128/IAI.69.11.6813-6822.2001 PMC100059 11598054

[30] Kim H Yoo SJ Kang HA. Yeast synthetic biology for the production of recombinant therapeutic proteins. FEMS Yeast Res. (2015) 15:1–16. doi: 10.1111/1567-1364.12195 25130199

[31] Shi S Valle-Rodriguez JO Siewers V Nielsen J. Engineering of chromosomal wax ester synthase integrated saccharomyces cerevisiae mutants for improved biosynthesis of fatty acid ethyl esters. Biotechnol Bioeng. (2014) 111:1740–7. doi: 10.1002/bit.25234 24752598

[32] Song X Liu Q Mao J Wu Y Li Y Gao K. Pot1-mediated delta-integration strategy for high-copy, stable expression of heterologous proteins in saccharomyces cerevisiae. FEMS Yeast Res. (2017) 17:6–064. doi: 10.1093/femsyr/fox064 28922845

[33] Xia T Yang H Guo Y Guo T Xin L Jiang Y. Human dendritic cell targeting peptide can be targeted to porcine dendritic cells to improve antigen capture efficiency to stimulate stronger immune response. Front Immunol. (2022) 13:950597. doi: 10.3389/fimmu.2022.950597 36059519 PMC9437479

[34] Owen JL Sahay B Mohamadzadeh M. New generation of oral mucosal vaccines targeting dendritic cells. Curr Opin Chem Biol. (2013) 17:918–24. doi: 10.1016/j.cbpa.2013.06.013 PMC3855095 23835515

[35] Banchereau J Steinman RM. Dendritic cells and the control of immunity. Nature. (1998) 392:245–52. doi: 10.1038/32588 9521319

[36] Brayden DJ Jepson MA Baird AW. Keynote review: intestinal peyer’s patch M cells and oral vaccine targeting. Drug Discov Today. (2005) 10:1145–57. doi: 10.1016/S1359-6446(05)03536-1 16182207

[37] Kunisawa J Kurashima Y Kiyono H. Gut-associated lymphoid tissues for the development of oral vaccines. Adv Drug Deliv Rev. (2012) 64:523–30. doi: 10.1016/j.addr.2011.07.003 21827802

[38] Li M Wang Y Sun Y Cui H Zhu SJ Qiu HJ. Mucosal vaccines: strategies and challenges. Immunol Lett. (2020) 217:116–25. doi: 10.1016/j.imlet.2019.10.013 31669546

[39] Colonna M Pulendran B Iwasaki A. Dendritic cells at the host-pathogen interface. Nat Immunol. (2006) 7:117–20. doi: 10.1038/ni0206-117 16424884

[40] Kim HJ Kim HO Lee K Baek EJ Kim HS. Two-step maturation of immature dcs with proinflammatory cytokine cocktail and poly(I:C) enhances migratory and T cell stimulatory capacity. Vaccine. (2010) 28:2877–86. doi: 10.1016/j.vaccine.2010.01.061 20156531

[41] Milling S Yrlid U Cerovic V MacPherson G. Subsets of migrating intestinal dendritic cells. Immunol Rev. (2010) 234:259–67. doi: 10.1111/j.0105-2896.2009.00866.x 20193024

[42] Liu S Luo Y Wang Y Li S Zhao Z Bi Y. Cryo-em structure of the african swine fever virus. Cell Host Microbe. (2019) 26:836–43 e3. doi: 10.1016/j.chom.2019.11.004 31787524

[43] Urbano AC Ferreira F. African swine fever control and prevention: an update on vaccine development. Emerg Microbes Infect. (2022) 11:2021–33. doi: 10.1080/22221751.2022.2108342 PMC9423837 35912875

[44] Dixon LK Islam M Nash R Reis AL. African swine fever virus evasion of host defences. Virus Res. (2019) 266:25–33. doi: 10.1016/j.virusres.2019.04.002 30959069 PMC6505686

[45] Zhao Y Yang J Niu Q Wang J Jing M Guan G. Identification and characterization of nanobodies from a phage display library and their application in an immunoassay for the sensitive detection of african swine fever virus. J Clin Microbiol. (2023) 61:e0119722. doi: 10.1128/jcm.01197-22 37154731 PMC10281114

[46] Cherf GM Cochran JR. Applications of yeast surface display for protein engineering. Methods Mol Biol. (2015) 1319:155–75. doi: 10.1007/978-1-4939-2748-7_8 PMC4544684 26060074

[47] Ou B Yang Y Lv H Lin X Zhang M. Current progress and challenges in the study of adjuvants for oral vaccines. BioDrugs. (2023) 37:143–80. doi: 10.1007/s40259-022-00575-1 PMC9821375 36607488

[48] Jin YB Yang WT Shi CW Feng B Huang KY Zhao GX. Immune responses induced by recombinant lactobacillus plantarum expressing the spike protein derived from transmissible gastroenteritis virus in piglets. Appl Microbiol Biotechnol. (2018) 102:8403–17. doi: 10.1007/s00253-018-9205-0 PMC7080080 30022263

[49] Holmgren J Czerkinsky C. Mucosal immunity and vaccines. Nat Med. (2005) 11:S45–53. doi: 10.1038/nm1213 15812489

[50] Sanchez EG Riera E Nogal M Gallardo C Fernandez P Bello-Morales R. Phenotyping and susceptibility of established porcine cells lines to african swine fever virus infection and viral production. Sci Rep. (2017) 7:10369. doi: 10.1038/s41598-017-09948-x 28871180 PMC5583235

[51] Truong QL Wang L Nguyen TA Nguyen HT Tran SD Vu AT. A cell-adapted live-attenuated vaccine candidate protects pigs against the homologous strain vnua-asfv-05l1, a representative strain of the contemporary pandemic african swine fever virus. Viruses. (2023) 15:10–2089. doi: 10.3390/v15102089 PMC10612049 37896866

[52] Niederwerder MC Stoian AMM Rowland RRR Dritz SS Petrovan V Constance LA. Infectious dose of african swine fever virus when consumed naturally in liquid or feed. Emerg Infect Dis. (2019) 25:891–7. doi: 10.3201/eid2505.181495 PMC6478231 30761988

[53] Lu S Li F Chen Q Wu J Duan J Lei X. Rapid detection of african swine fever virus using cas12a-based portable paper diagnostics. Cell Discov. (2020) 6:18. doi: 10.1038/s41421-020-0151-5 32284877 PMC7136273

[54] Perez-Nunez D Sunwoo SY Sanchez EG Haley N Garcia-Belmonte R Nogal M. Evaluation of a viral DNA-protein immunization strategy against african swine fever in domestic pigs. Vet Immunol Immunopathol. (2019) 208:34–43. doi: 10.1016/j.vetimm.2018.11.018 30712790

[55] Austriaco N. Yeast oral vaccines against infectious diseases. Front Microbiol. (2023) 14:1150412. doi: 10.3389/fmicb.2023.1150412 37138614 PMC10149678

[56] Jin Z Gao S Cui X Sun D Zhao K. Adjuvants and delivery systems based on polymeric nanoparticles for mucosal vaccines. Int J Pharm. (2019) 572:118731. doi: 10.1016/j.ijpharm.2019.118731 31669213

[57] Khatib AM Ali M von Seidlein L Kim DR Hashim R Reyburn R. Effectiveness of an oral cholera vaccine in zanzibar: findings from a mass vaccination campaign and observational cohort study. Lancet Infect Dis. (2012) 12:837–44. doi: 10.1016/S1473-3099(12)70196-2 22954655

[58] Quan FS Compans RW Kang SM. Oral vaccination with inactivated influenza vaccine induces cross-protective immunity. Vaccine. (2012) 30:180–8. doi: 10.1016/j.vaccine.2011.11.028 PMC5555299 22107852

[59] Slomski A. Novel oral polio vaccine safely induces antibodies among vaccine-naive infants. JAMA. (2023) 329:279. doi: 10.1001/jama.2022.25137 36692562

[60] Crunkhorn S. A bacteria-derived oral tumour vaccine. Nat Rev Drug Discov. (2022) 21:494. doi: 10.1038/d41573-022-00100-7 35676336

[61] Borca MV Ramirez-Medina E Silva E Vuono E Rai A Pruitt S. Asfv-G-ΔI177l as an effective oral nasal vaccine against the eurasia strain of Africa swine fever. Viruses. (2021) 13:5–765. doi: 10.3390/v13050765 PMC8146859 33925435

[62] Chen C Hua D Shi J Tan Z Zhu M Tan K. Porcine immunoglobulin fc fused P30/P54 protein of african swine fever virus displaying on surface of S. Cerevisiae elicit strong antibody production in swine. Virol Sin. (2021) 36:207–19. doi: 10.1007/s12250-020-00278-3 PMC8087729 32915442

[63] Milligan GN Dudley-McClain KL Chu CF Young CG. Efficacy of genital T cell responses to herpes simplex virus type 2 resulting from immunization of the nasal mucosa. Virology. (2004) 318:507–15. doi: 10.1016/j.virol.2003.10.010 14972519

[64] Zhai L Yadav R Kunda NK Anderson D Bruckner E Miller EK. Oral immunization with bacteriophage ms2-L2 vlps protects against oral and genital infection with multiple hpv types associated with head & Neck cancers and cervical cancer. Antiviral Res. (2019) 166:56–65. doi: 10.1016/j.antiviral.2019.03.012 30926288 PMC6538018

[65] Crozat K Tamoutounour S Vu Manh TP Fossum E Luche H Ardouin L. Cutting edge: expression of xcr1 defines mouse lymphoid-tissue resident and migratory dendritic cells of the cd8alpha+ Type. J Immunol. (2011) 187:4411–5. doi: 10.4049/jimmunol.1101717 21948982

[66] Haniffa M Shin A Bigley V McGovern N Teo P See P. Human tissues contain cd141hi cross-presenting dendritic cells with functional homology to mouse cd103+ Nonlymphoid dendritic cells. Immunity. (2012) 37:60–73. doi: 10.1016/j.immuni.2012.04.012 22795876 PMC3476529

[67] Crozat K Guiton R Contreras V Feuillet V Dutertre CA Ventre E. The xc chemokine receptor 1 is a conserved selective marker of mammalian cells homologous to mouse cd8alpha+ Dendritic cells. J Exp Med. (2010) 207:1283–92. doi: 10.1084/jem.20100223 PMC2882835 20479118

[68] Deloizy C Bouguyon E Fossum E Sebo P Osicka R Bole A. Expanding the tools for identifying mononuclear phagocyte subsets in swine: reagents to porcine cd11c and xcr1. Dev Comp Immunol. (2016) 65:31–40. doi: 10.1016/j.dci.2016.06.015 27345169

[69] Song J Wang M Zhou L Tian P Sun Z Sun J. A candidate nanoparticle vaccine comprised of multiple epitopes of the african swine fever virus elicits a robust immune response. J Nanobiotechnol. (2023) 21:424. doi: 10.1186/s12951-023-02210-9 PMC10647103 37964304

[70] Rajkhowa S Sonowal J Pegu SR Sanger GS Deb R Das PJ. Natural co-infection of pigs with african swine fever virus and porcine reproductive and respiratory syndrome virus in India. Braz J Microbiol. (2023) 55:1017–22. doi: 10.1007/s42770-023-01203-y PMC10920511 38041718

[71] Liu H Zou J Liu R Chen J Li X Zheng H. Development of a taqman-probe-based multiplex real-time pcr for the simultaneous detection of african swine fever virus, porcine circovirus 2, and pseudorabies virus in east China from 2020 to 2022. Vet Sci. (2023) 10:2–106. doi: 10.3390/vetsci10020106 PMC9964870 36851410

[72] Jiao P Wang S Fan W Zhang H Yin H Shang Y. Recombinant porcine interferon cocktail delays the onset and lessens the severity of african swine fever. Antiviral Res. (2023) 215:105644. doi: 10.1016/j.antiviral.2023.105644 37244381

[73] Wang T Sun Y Huang S Qiu HJ. Multifaceted immune responses to african swine fever virus: implications for vaccine development. Vet Microbiol. (2020) 249:108832. doi: 10.1016/j.vetmic.2020.108832 32932135

[74] Teklue T Sun Y Abid M Luo Y Qiu HJ. Current status and evolving approaches to african swine fever vaccine development. Transbound Emerg Dis. (2020) 67:529–42. doi: 10.1111/tbed.13364 31538406

[75] Dang W Li T Xu F Wang Y Yang F Zheng H. Establishment of a crispr/cas9 knockout library for screening type I interferon-inducible antiviral effectors in pig cells. Front Immunol. (2022) 13:1016545. doi: 10.3389/fimmu.2022.1016545 36505425 PMC9732717

[76] Liu Y Zhang X Liu Z Huang L Jia W Lian X. Toosendanin suppresses african swine fever virus replication through upregulating interferon regulatory factor 1 in porcine alveolar macrophage cultures. Front Microbiol. (2022) 13:970501. doi: 10.3389/fmicb.2022.970501 36110293 PMC9468581

[77] Zheng Y Li S Li SH Yu S Wang Q Zhang K. Transcriptome profiling in swine macrophages infected with african swine fever virus at single-cell resolution. Proc Natl Acad Sci U.S.A. (2022) 119:e2201288119. doi: 10.1073/pnas.2201288119 35507870 PMC9171760

[78] Zhang H Zhao S Zhang H Qin Z Shan H Cai X. Vaccines for african swine fever: an update. Front Microbiol. (2023) 14:1139494. doi: 10.3389/fmicb.2023.1139494 37180260 PMC10173882

[79] Sunwoo SY Perez-Nunez D Morozov I Sanchez EG Gaudreault NN Trujillo JD. DNA-protein vaccination strategy does not protect from challenge with african swine fever virus Armenia 2007 strain. Vaccines (Basel). (2019) 7:1–12. doi: 10.3390/vaccines7010012 PMC6466342 30696015

