# Supplementary material for: Saccharomyces cerevisiae oral immunization in mice using multi-antigen of the African swine fever virus elicits a robust immune response
Source: Front Immunol. 2024 Apr 29;15:1373656. doi: 10.3389/fimmu.2024.1373656 (PMC11089227; doi:10.3389/fimmu.2024.1373656)
Supplement: Supplementary file 1 [file DataSheet_1.docx]

Supplementary Figures

##
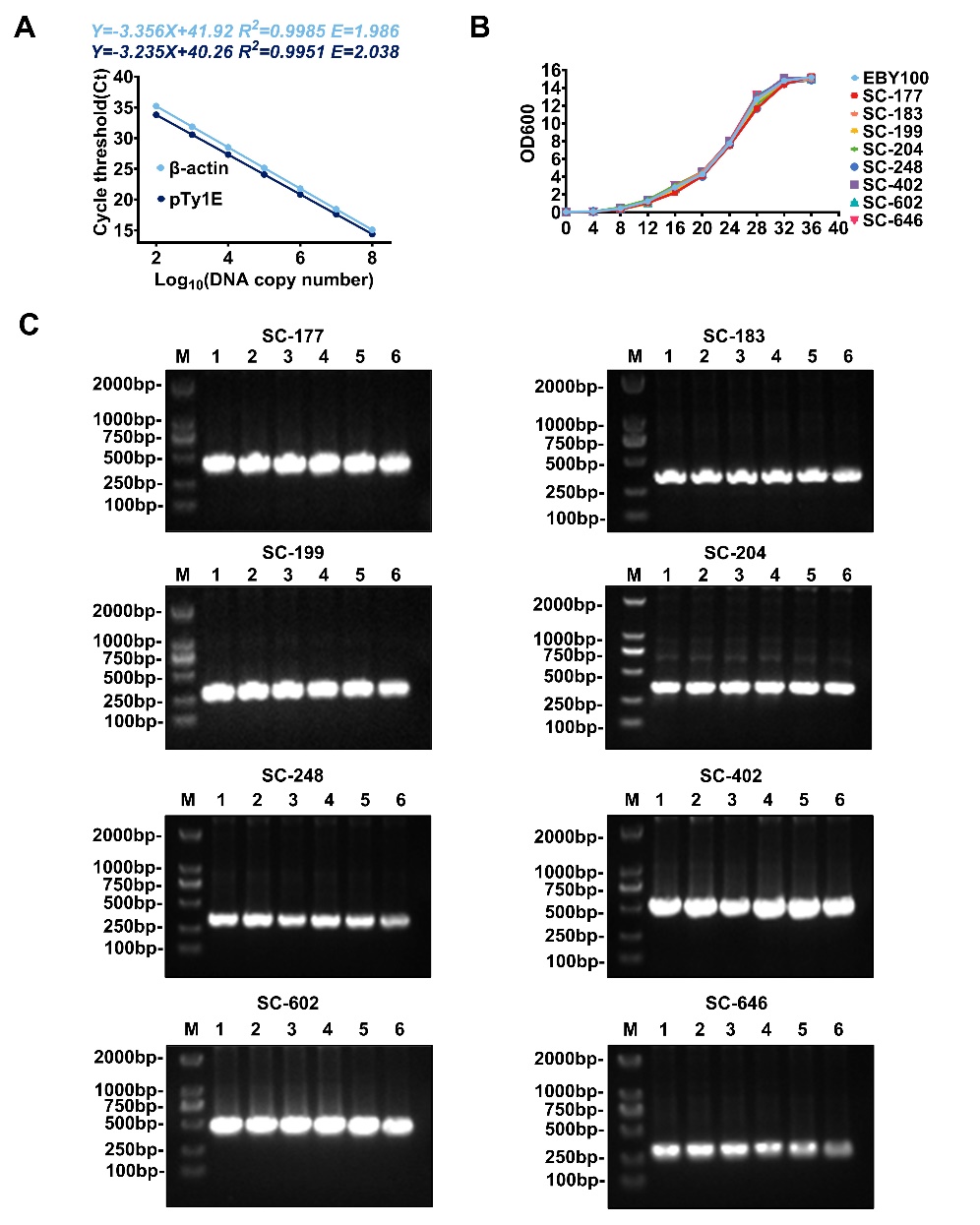


**Supplementary Figure 1. The recombinant strains of Saccharomyces cerevisiae were constructed using the δ-integration strategy to achieve high copy number of ASFV antigen cocktails. (A)**The standard curve of the recombinant plasmid was obtained by a qPCR assay. Of the recombinant plasmid, 10^8^–10^2^copies/ml were used as the template for the reaction. A standard curve of the correlation between DNA copy number and qPCR cycle threshold (Ct) value was constructed using GraphPad Prism version 9.4.1.681. R^2^ represents the degree of fit of the regression line. The slope of the standard curve represents the amplification efficiency (E), through the following formula E =10 (‒1/slope). **(B)** Growth curves of EBY100 and recombinant Saccharomyces cerevisiae strains. Growth progression was monitored by measurement of the optical density at 600 nm (OD 600). **(C)** Genetic identity and genetic stability of the recombinant saccharomyces cerevisiae strains were assessed via polymerase chain reaction (PCR) analysis of the genomic DNA. M is for DNA size marker. Strains from subcultures 1, 10, 20, 30, 40, and 50 (designated as 1, 2, 3, 4, 5, and 6 respectively) were utilized as seeds for cultivation and subsequent analysis.


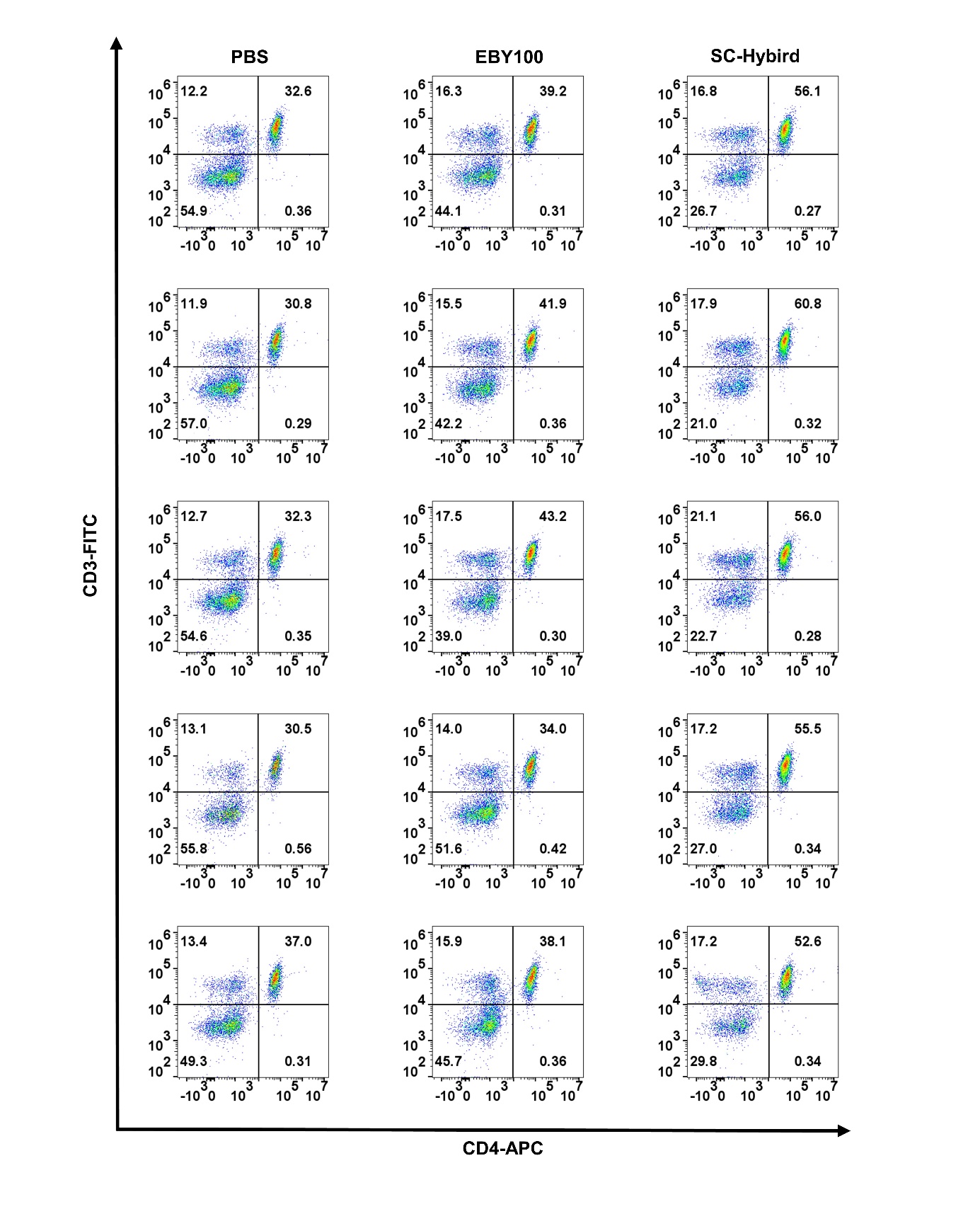


**Supplementary Figure 2. The percentages of CD3 + and CD4 + T lymphocytes in the purified splenocytes were determined by flow cytometry.**

**
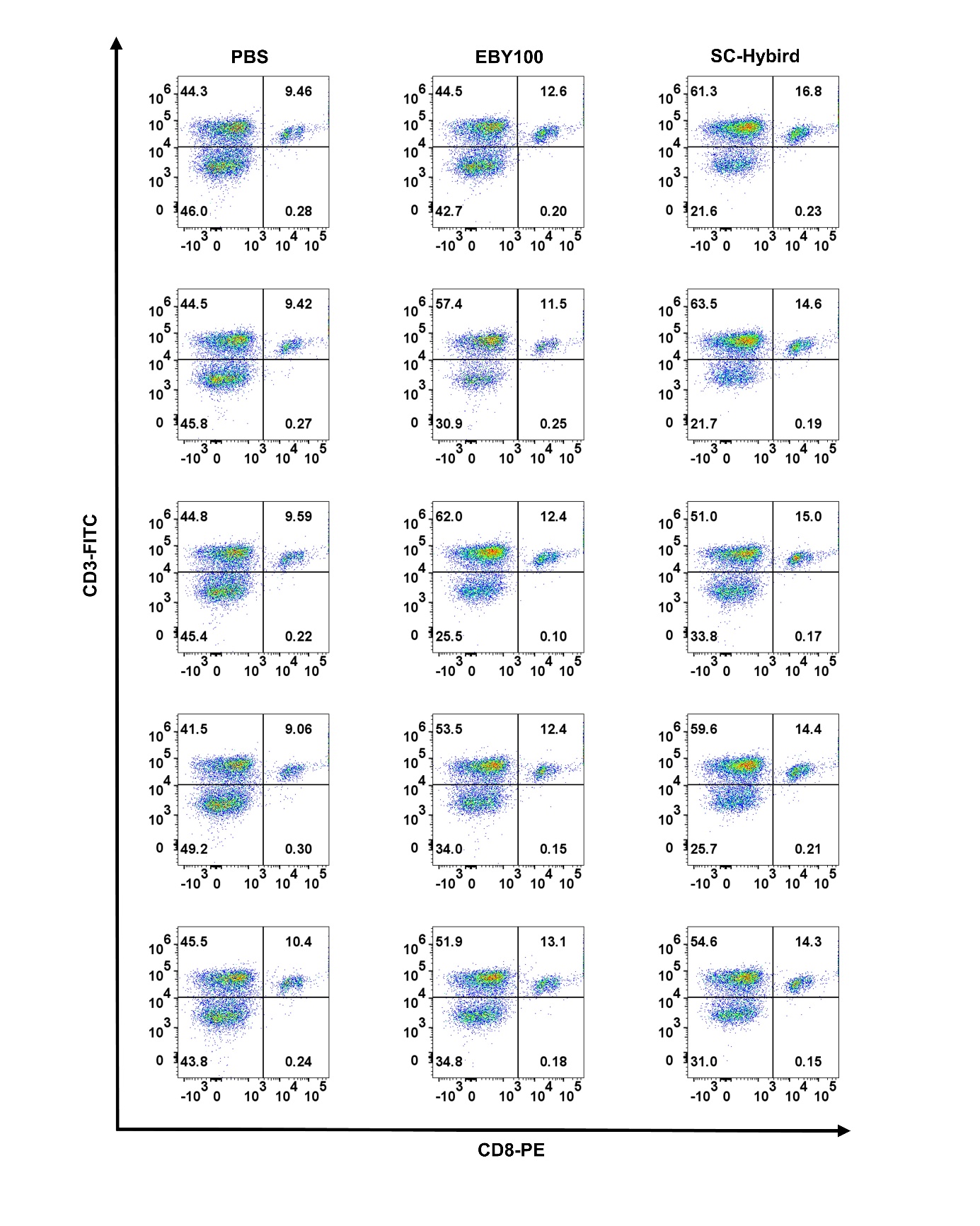
**

**Supplementary Figure 3. The percentages of CD3 + and CD8 + T lymphocytes in the purified splenocytes were determined by flow cytometry.**
